# (Acetoxy)(2-methylphenyl)methyl acetate

**DOI:** 10.1107/S1600536811028625

**Published:** 2011-07-23

**Authors:** J. Kanchanadevi, G. Anbalagan, V. Saravanan, A. K. Mohanakrishnan, V. Manivannan

**Affiliations:** a Department of Physics, Velammal Institute of Technology, Panchetty, Chennai 601204, India; b Department of Physics, Presidency College (Autonomous), Chennai 600 005, India; c Department of Organic Chemistry, University of Madras, Guindy Campus, Chennai 600 025, India; d Department of Research and Development, PRIST University, Vallam, Thanjavur 613 403, Tamil Nadu, India

## Abstract

In the title compound, C_12_H_14_O_4_, the two acet­oxy groups are inclined by 57.92 (5)° and 62.71 (6)° to the benzene ring. An inter­molecular C—H⋯O inter­action involving the two acet­oxy groups generates a centrosymmetric dimer *via* an *R* ^2^_2_(16) ring motif.

## Related literature

For the structure of the 4-methyl isomer, see: Rajnikant *et al.* (2009 ▶). For graph-set notation, see: Bernstein *et al.* (1995 ▶)
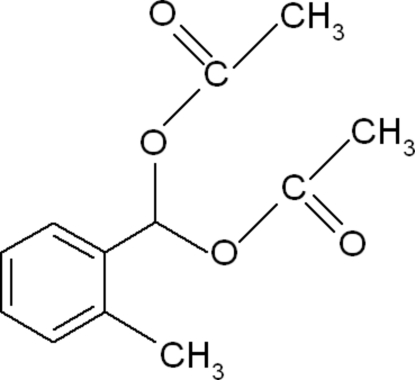


## Experimental

### 

#### Crystal data

C_12_H_14_O_4_
*M* *_r_* = 222.23
Monoclinic, 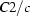

*a* = 15.757 (5) Å
*b* = 7.564 (5) Å
*c* = 19.886 (5) Å
β = 99.17 (5)°
*V* = 2339.8 (18) Å^3^
*Z* = 8
Mo *K*α radiation
μ = 0.10 mm^−1^
*T* = 295 K
0.25 × 0.20 × 0.15 mm

#### Data collection

Bruker Kappa APEXII CCD diffractometer
Absorption correction: multi-scan (*SADABS*; Sheldrick, 1996 ▶) *T*_min_ = 0.950, *T*_max_ = 0.975
12571 measured reflections
2414 independent reflections
1856 reflections with *I* > 2σ(*I*)
*R*_int_ = 0.027

#### Refinement

*R*[*F* ^2^ > 2σ(*F* ^2^)] = 0.041
*wR*(*F* ^2^) = 0.125
*S* = 1.05
2414 reflections
149 parameters
H-atom parameters constrained
Δρ_max_ = 0.20 e Å^−3^
Δρ_min_ = −0.19 e Å^−3^

### 

Data collection: *APEX2* (Bruker, 2004 ▶); cell refinement: *SAINT* (Bruker, 2004 ▶); data reduction: *SAINT*; program(s) used to solve structure: *SHELXS97* (Sheldrick, 2008 ▶); program(s) used to refine structure: *SHELXL97* (Sheldrick, 2008 ▶); molecular graphics: *PLATON* (Spek, 2009 ▶); software used to prepare material for publication: *SHELXL97*.

## Supplementary Material

Crystal structure: contains datablock(s) I. DOI: 10.1107/S1600536811028625/gk2393sup1.cif

Structure factors: contains datablock(s) I. DOI: 10.1107/S1600536811028625/gk2393Isup2.hkl

Supplementary material file. DOI: 10.1107/S1600536811028625/gk2393Isup3.cml

Additional supplementary materials:  crystallographic information; 3D view; checkCIF report

## Figures and Tables

**Table 1:** Hydrogen-bond geometry (Å, °)

*D*—H⋯*A*	*D*—H	H⋯*A*	*D*⋯*A*	*D*—H⋯*A*
C9—H9*A*⋯O4^i^	0.96	2.50	3.425 (3)	161

Symmetry code: (i) 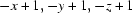.

**Fig. 1.**
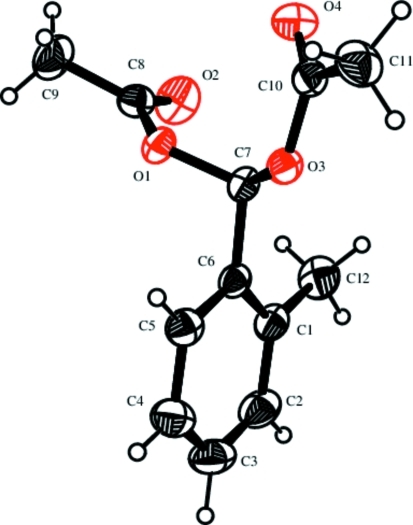
The molecular structure of the title compound, with atom labels and 30% probability displacement ellipsoids for non-H atoms.

**Fig. 2.**
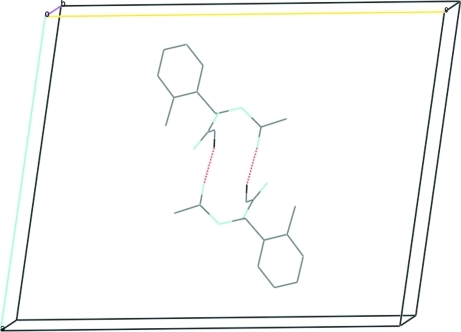
The R22(16) ring set motif of the title compound, viewed down the b axis. Hydrogen bonds are shown as dashed lines (hydrogen atoms have been omitted).

## supplementary crystallographic information

### Comment

The geometric parameters of the title molecule (Fig. 1) agree well with similar structure (Rajnikant *et al.*, 2009). Intermolecular C—H···O interaction involving the two acetoxy groups generates a centrosymmetric dimer *via R*^2^_2_(16) ring motif, Fig. 2 (Bernstein *et al.*, 1995).

### Experimental

To a solution of 2-methylbenzaldehyde (5 g, 41.61 mmol) in dry acetic anhydride (25 ml) anhydrous indium bromide (0.147 g, 0.416 mmol) was added. It was then stirred at room temperature for 4 h under nitrogen atmosphere. The reaction mixture was then poured over crushed ice (300 g). The solid obtained was filtered and washed thoroughly with water and the product was recrystallized from methanol to give pure product as a colorless solid with a yield of 82% and melting point 333 K.

### Refinement

H atoms were positioned geometrically and refined using riding model with C—H = 0.93 Å and *U*_iso_(H) = 1.2U_eq_(C) for aromatic C—H, C—H = 0.98 Å and *U*_iso_(H) = 1.2U_eq(_C) for methine C—H, C—H = 0.96 Å and *U*_iso_(H) = 1.5U_eq_(C) for methyl group.

### Crystal data

**Table:**

C_12_H_14_O_4_	*F*(000) = 944
*M**_r_* = 222.23	*D*_x_ = 1.262 Mg m^−^^3^
Monoclinic, *C*2/*c*	Mo *K*α radiation, λ = 0.71073 Å
Hall symbol: -C 2yc	Cell parameters from 7598 reflections
*a* = 15.757 (5) Å	θ = 2.1–26.5°
*b* = 7.564 (5) Å	µ = 0.10 mm^−^^1^
*c* = 19.886 (5) Å	*T* = 295 K
β = 99.17 (5)°	Block, colourless
*V* = 2339.8 (18) Å^3^	0.25 × 0.20 × 0.15 mm
*Z* = 8	

### Data collection

**Table:**

Bruker Kappa APEXII CCD diffractometer	2414 independent reflections
Radiation source: fine-focus sealed tube	1856 reflections with *I* > 2σ(*I*)
graphite	*R*_int_ = 0.027
ω and φ scans	θ_max_ = 26.5°, θ_min_ = 2.1°
Absorption correction: multi-scan (*SADABS*; Sheldrick, 1996)	*h* = −13→19
*T*_min_ = 0.950, *T*_max_ = 0.975	*k* = −9→8
12571 measured reflections	*l* = −24→24

### Refinement

**Table:**

Refinement on *F*^2^	Secondary atom site location: difference Fourier map
Least-squares matrix: full	Hydrogen site location: inferred from neighbouring sites
*R*[*F*^2^ > 2σ(*F*^2^)] = 0.041	H-atom parameters constrained
*wR*(*F*^2^) = 0.125	*w* = 1/[σ^2^(*F*_o_^2^) + (0.0609*P*)^2^ + 0.9232*P*] where *P* = (*F*_o_^2^ + 2*F*_c_^2^)/3
*S* = 1.05	(Δ/σ)_max_ = 0.001
2414 reflections	Δρ_max_ = 0.20 e Å^−^^3^
149 parameters	Δρ_min_ = −0.19 e Å^−^^3^
0 restraints	Extinction correction: *SHELXL*, Fc^*^=kFc[1+0.001xFc^2^λ^3^/sin(2θ)]^-1/4^
Primary atom site location: structure-invariant direct methods	Extinction coefficient: 0.0066 (8)

### Fractional atomic coordinates and isotropic or equivalent isotropic displacement parameters (Å^2^)

**Table:**

	*x*	*y*	*z*	*U*_iso_*/*U*_eq_	
C1	0.70247 (11)	−0.0332 (2)	0.68559 (8)	0.0539 (4)	
C2	0.77083 (15)	−0.0812 (3)	0.73557 (9)	0.0703 (6)	
H2	0.7592	−0.1354	0.7751	0.084*	
C3	0.85476 (14)	−0.0510 (3)	0.72841 (10)	0.0744 (6)	
H3	0.8990	−0.0842	0.7628	0.089*	
C4	0.87350 (12)	0.0281 (3)	0.67050 (10)	0.0670 (5)	
H4	0.9303	0.0492	0.6654	0.080*	
C5	0.80723 (10)	0.0760 (2)	0.61999 (9)	0.0530 (4)	
H5	0.8196	0.1295	0.5806	0.064*	
C6	0.72234 (10)	0.04578 (19)	0.62698 (7)	0.0447 (4)	
C7	0.65391 (10)	0.10316 (19)	0.56986 (7)	0.0434 (4)	
H7	0.5963	0.0783	0.5802	0.052*	
C8	0.60492 (10)	0.3993 (2)	0.57295 (8)	0.0513 (4)	
C9	0.61840 (14)	0.5811 (2)	0.54922 (12)	0.0794 (6)	
H9A	0.5646	0.6435	0.5424	0.119*	
H9B	0.6407	0.5762	0.5071	0.119*	
H9C	0.6586	0.6416	0.5828	0.119*	
C10	0.60738 (10)	0.0292 (2)	0.45432 (8)	0.0494 (4)	
C11	0.62941 (13)	−0.0747 (3)	0.39646 (10)	0.0717 (5)	
H11A	0.5777	−0.1068	0.3666	0.108*	
H11B	0.6598	−0.1798	0.4133	0.108*	
H11C	0.6651	−0.0050	0.3719	0.108*	
C12	0.61143 (13)	−0.0643 (3)	0.69723 (10)	0.0740 (6)	
H12A	0.6119	−0.1331	0.7379	0.111*	
H12B	0.5804	−0.1269	0.6591	0.111*	
H12C	0.5840	0.0472	0.7022	0.111*	
O1	0.66431 (6)	0.28750 (13)	0.55632 (5)	0.0477 (3)	
O2	0.54980 (9)	0.35382 (19)	0.60388 (8)	0.0794 (4)	
O3	0.66880 (7)	0.01209 (13)	0.51011 (5)	0.0469 (3)	
O4	0.54496 (8)	0.11891 (18)	0.45344 (6)	0.0649 (4)	

### Atomic displacement parameters (Å^2^)

**Table:**

	*U*^11^	*U*^22^	*U*^33^	*U*^12^	*U*^13^	*U*^23^
C1	0.0757 (11)	0.0441 (9)	0.0415 (9)	0.0013 (8)	0.0083 (8)	0.0000 (7)
C2	0.1023 (16)	0.0611 (11)	0.0444 (10)	0.0105 (11)	0.0019 (9)	0.0072 (8)
C3	0.0860 (14)	0.0725 (13)	0.0567 (12)	0.0227 (11)	−0.0132 (10)	−0.0030 (10)
C4	0.0614 (10)	0.0700 (12)	0.0659 (12)	0.0116 (9)	−0.0017 (9)	−0.0120 (10)
C5	0.0596 (10)	0.0499 (9)	0.0496 (9)	0.0049 (7)	0.0089 (7)	−0.0031 (7)
C6	0.0590 (9)	0.0351 (7)	0.0394 (8)	0.0033 (6)	0.0060 (6)	−0.0028 (6)
C7	0.0533 (8)	0.0358 (8)	0.0423 (8)	0.0010 (6)	0.0119 (6)	0.0014 (6)
C8	0.0531 (9)	0.0507 (9)	0.0482 (9)	0.0113 (7)	0.0023 (7)	−0.0071 (7)
C9	0.0886 (14)	0.0432 (10)	0.1066 (17)	0.0173 (10)	0.0163 (12)	0.0014 (10)
C10	0.0558 (9)	0.0470 (9)	0.0445 (9)	−0.0060 (7)	0.0055 (7)	0.0033 (7)
C11	0.0824 (13)	0.0797 (13)	0.0508 (10)	−0.0020 (10)	0.0039 (9)	−0.0145 (9)
C12	0.0912 (14)	0.0805 (13)	0.0538 (11)	−0.0122 (11)	0.0222 (10)	0.0127 (10)
O1	0.0520 (6)	0.0355 (6)	0.0573 (7)	0.0054 (4)	0.0135 (5)	0.0036 (5)
O2	0.0832 (9)	0.0732 (9)	0.0908 (10)	0.0161 (7)	0.0418 (8)	−0.0052 (8)
O3	0.0573 (6)	0.0417 (6)	0.0407 (6)	0.0042 (4)	0.0053 (5)	−0.0033 (4)
O4	0.0595 (7)	0.0741 (9)	0.0586 (7)	0.0104 (6)	0.0021 (6)	0.0037 (6)

### Geometric parameters (Å, °)

**Table:**

C1—C6	1.389 (2)	C8—O2	1.192 (2)
C1—C2	1.392 (3)	C8—O1	1.3413 (19)
C1—C12	1.508 (3)	C8—C9	1.480 (3)
C2—C3	1.372 (3)	C9—H9A	0.9600
C2—H2	0.9300	C9—H9B	0.9600
C3—C4	1.371 (3)	C9—H9C	0.9600
C3—H3	0.9300	C10—O4	1.1929 (19)
C4—C5	1.377 (2)	C10—O3	1.3572 (19)
C4—H4	0.9300	C10—C11	1.480 (3)
C5—C6	1.386 (2)	C11—H11A	0.9600
C5—H5	0.9300	C11—H11B	0.9600
C6—C7	1.500 (2)	C11—H11C	0.9600
C7—O3	1.4248 (18)	C12—H12A	0.9600
C7—O1	1.434 (2)	C12—H12B	0.9600
C7—H7	0.9800	C12—H12C	0.9600
			
C6—C1—C2	117.32 (17)	O2—C8—C9	125.89 (16)
C6—C1—C12	122.91 (15)	O1—C8—C9	111.48 (16)
C2—C1—C12	119.76 (17)	C8—C9—H9A	109.5
C3—C2—C1	122.11 (18)	C8—C9—H9B	109.5
C3—C2—H2	118.9	H9A—C9—H9B	109.5
C1—C2—H2	118.9	C8—C9—H9C	109.5
C4—C3—C2	120.01 (17)	H9A—C9—H9C	109.5
C4—C3—H3	120.0	H9B—C9—H9C	109.5
C2—C3—H3	120.0	O4—C10—O3	123.03 (15)
C3—C4—C5	119.19 (19)	O4—C10—C11	125.86 (16)
C3—C4—H4	120.4	O3—C10—C11	111.10 (15)
C5—C4—H4	120.4	C10—C11—H11A	109.5
C4—C5—C6	121.02 (17)	C10—C11—H11B	109.5
C4—C5—H5	119.5	H11A—C11—H11B	109.5
C6—C5—H5	119.5	C10—C11—H11C	109.5
C5—C6—C1	120.36 (15)	H11A—C11—H11C	109.5
C5—C6—C7	117.72 (14)	H11B—C11—H11C	109.5
C1—C6—C7	121.92 (14)	C1—C12—H12A	109.5
O3—C7—O1	105.94 (11)	C1—C12—H12B	109.5
O3—C7—C6	107.31 (12)	H12A—C12—H12B	109.5
O1—C7—C6	109.49 (12)	C1—C12—H12C	109.5
O3—C7—H7	111.3	H12A—C12—H12C	109.5
O1—C7—H7	111.3	H12B—C12—H12C	109.5
C6—C7—H7	111.3	C8—O1—C7	117.51 (13)
O2—C8—O1	122.63 (16)	C10—O3—C7	116.42 (12)
			
C6—C1—C2—C3	0.7 (3)	C1—C6—C7—O3	−121.51 (15)
C12—C1—C2—C3	−178.03 (19)	C5—C6—C7—O1	−55.29 (17)
C1—C2—C3—C4	−0.3 (3)	C1—C6—C7—O1	123.95 (15)
C2—C3—C4—C5	−0.1 (3)	O2—C8—O1—C7	7.3 (2)
C3—C4—C5—C6	0.1 (3)	C9—C8—O1—C7	−173.66 (14)
C4—C5—C6—C1	0.3 (2)	O3—C7—O1—C8	134.50 (12)
C4—C5—C6—C7	179.53 (15)	C6—C7—O1—C8	−110.08 (14)
C2—C1—C6—C5	−0.7 (2)	O4—C10—O3—C7	2.1 (2)
C12—C1—C6—C5	177.99 (17)	C11—C10—O3—C7	−178.64 (14)
C2—C1—C6—C7	−179.89 (15)	O1—C7—O3—C10	−71.00 (15)
C12—C1—C6—C7	−1.2 (2)	C6—C7—O3—C10	172.10 (12)
C5—C6—C7—O3	59.25 (17)		

### Hydrogen-bond geometry (Å, °)

**Table:**

*D*—H···*A*	*D*—H	H···*A*	*D*···*A*	*D*—H···*A*
C9—H9A···O4^i^	0.96	2.50	3.425 (3)	161

Symmetry codes: (i) −*x*+1, −*y*+1, −*z*+1.
